# Neurons promote encephalitogenic CD4^+^ lymphocyte infiltration in experimental autoimmune encephalomyelitis

**DOI:** 10.1038/s41598-020-64363-z

**Published:** 2020-04-30

**Authors:** Yuki Nakazato, Yuki Fujita, Masamitsu Nakazato, Toshihide Yamashita

**Affiliations:** 1 Department of Molecular Neuroscience, Graduate School of Medicine, Osaka University, Osaka, Japan; 2 WPI Immunology Frontier Research Center, Osaka University, Osaka, Japan; 3 Department of Internal Medicine, Division of Neurology, Respirology, Endocrinology, and Metabolism, Department of Internal Medicine, Faculty of Medicine, University of Miyazaki, Miyazaki, Japan; 4 Graduate School of Frontier Bioscience, Osaka University, Osaka, Japan; 5 Department of Neuro-Medical Science, Graduate School of Medicine, Osaka University, Osaka, Japan

**Keywords:** Neurodegeneration, Neuroimmunology

## Abstract

Multiple sclerosis (MS) is an autoimmune disease of the central nervous system characterized by neuroinflammation, leading to demyelination and axonal degeneration. Neuronal excitotoxity mediated by Ca^2+^/calmodulin-dependent protein kinase IIα (CaMKIIα) results in neuronal damage in experimental autoimmune encephalitis (EAE), an animal model of MS. Here, we define a critical role of excitatory neurons in the pathogenesis of CD4^+^ lymphocyte accumulation in EAE. We silenced the activity of excitatory neurons in a mouse model of targeted EAE using inhibitory designer receptors exclusively activated by designer drugs (DREADD) under a CaMKIIα promoter. Neuronal silencing mitigated clinical disease scores in EAE, reduced the expression of *c-fos, Tnfα*, *Ccl2*, and *Ccr2* mRNAs in targeted EAE lesions, and prevented the migration of CD4^+^ lymphocytes towards neurons. *Ccl2* shRNA treatment of targeted EAE suppressed the migration of CD4^+^ lymphocytes and alleviated the motor deficits of EAE. Our findings indicate that neuronal activation in EAE promotes the migration of CCR2^+^ CD4^+^ lymphocytes and that neuronal silencing with an inhibitory DREADD alleviates clinical and molecular markers of disease. Neuronal CCL2 is thought to be involved in promoting lymphocytes migration.

## Introduction

Multiple sclerosis (MS) is one of the major autoimmune diseases in the central nervous system (CNS), characterized by neuroinflammation with consequent demyelination and axonal degeneration^1^. Generation of T cells reactive to myelin proteins is a pathological hallmark of MS, and increased the migration of autoreactive T cells across the blood-brain barrier (BBB) heralds the beginning of the disease process^2,3^. Thus, several agents that aim to suppress the migration of autoreactive T cells into the CNS have been developed as potential therapies for MS^4^.

Chemokines and chemokine receptors are key players in the migration of T cells across the BBB^5^. C-C motif chemokine ligand 2 (CCL2), which binds solely to the C-C motif chemokine receptor type 2 (CCR2), regulates the migration and the activation of T cells, monocytes, NK cells, and basophils^6^. Neuronal CCL2 drives inflammatory monocyte infiltration into the brain in Theiler’s murine encephalomyelitis virus infection model^7^, and promotes phagocyte recruitment in the viral déjà vu model^8^. EAE, the most commonly used experimental animal model of MS^9^, exhibits the pathological alterations and clinical symptoms of MS, including motor deficits. In a mouse model of EAE, anti-CCL2 neutralizing antibody reduced mononuclear cell infiltration into the CNS and clinical severity^10^. Further, CCL2-knockout mice exhibited less severe EAE clinical manifestations^3^, and CCR2-knockout mice failed to develop EAE^11^. Therefore, the CCL2-CCR2 axis has been regarded as critical for the development of EAE. CCL2 is expressed by astrocytes and neurons among the parenchymal cells in the CNS^12^. While deletion of astrocytic CCL2 diminished the severity of clinical deficits in EAE^13^, the pathophysiological significance of neuronal CCL2 in EAE has not yet been elucidated. In EAE, neuronal excitotoxity mediated by glutamate-glutamate receptor signals, amplified by Ca^2+^/calmodulin-dependent protein kinase IIα (CaMKIIα), resulted in the development of neuronal injury^14–16^. Similarly, an increase in phosphorylated CaMKIIα (pCaMKIIα) has been observed in the dorsal horn of the spinal column in EAE by quantitative immunohistochemistry, with both small interfering RNA targeting CaMKIIα and CaMKIIαT286A point mutation reducing EAE clinical scores^16^.

Designer receptors exclusively activated by designer drugs (DREADD) can be used to regulate cell activity in a cell-type-specific fashion in freely moving animals^17,18^. The inhibitory DREADD gated by clozapine-N-oxide (CNO), a pharmacologically inert metabolite of the antipsychotic drug clozapine, silences cell activity by inhibitory G-protein (Gi) signaling^17,18^. By using an osmotic minipump to release CNO continuously, the inhibitory DREADD can exert its designated action for a stipulated duration^19^.

In the present study, we aimed to determine how neuronal silencing would vary the migration of CD4^+^ lymphocytes and the changes in clinical and molecular disease characteristics neuronal silencing would produce.

## Results

### Inhibitory DREADD attenuates motor deficits in targeted EAE

**Figure 1:**
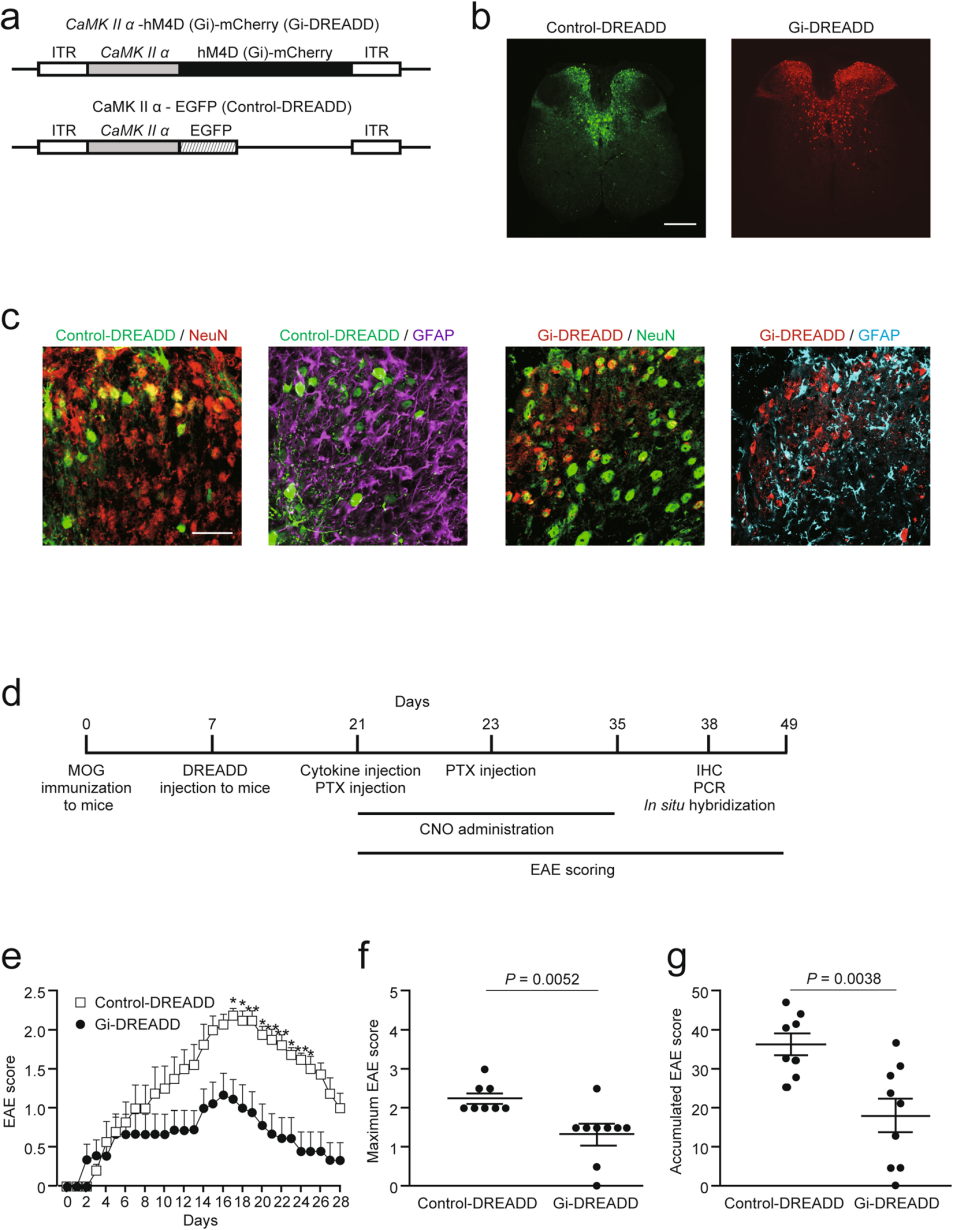
CNO-gated DREADD treatment suppressed EAE severity. (**a**) Schematic illustration of pAAV-CaMKIIα-hM4Di (Gi)-mCherry (Gi-DREADD) and pAAV-CaMKIIα-EGFP (control-DREADD). The AAV9 vector contains Gi- or control-DREADD under the CaMKIIα promoter. (**b**) Distribution of control- and Gi-DREADD at spinal level Th 8 in mice 14 days after injection of DREADD-carrying-AAV9. Scale bars: 150 μm. (**c**) Higher magnification images showing DREADD colocalization with NeuN or GFAP in the dorsal column of the Th 8 level spinal cord. NeuN (red) and GFAP (magenta) in control-DREADD (green) experiment. NeuN (green) and GFAP (cyan) in Gi-DREADD (red) experiment. Scale bar: 50 μm. (**d**) Experimental schema. Targeted EAE was induced with MOG_35–55_ peptide and local injection of TNFα and IFNγ. Seven days after MOG immunization, DREADD-carrying-AAV9 was injected at spinal level Th 9. EAE scores were assessed from day 0 to 28 after cytokine injection. (**e**) EAE scores throughout the 28 days (control: n = 8, Gi: n = 9; two-way ANOVA followed by the Sidak test). (**f)** Maximum and (**g**) accumulated EAE scores (control: n = 8, Gi: n = 9; Student’s *t-*test). Data are presented as mean ± sem.

To suppress neuronal activity, we injected an hM4Di-encoded adeno-associated virus 9 (AAV9) under a CaMKIIα promoter (CaMKIIα-hM4D [Gi]-mCherry, referred to herein as Gi-DREADD) at spinal level Th 9. A CaMKIIα AAV9 tagged with enhanced green fluorescent protein (EGFP; control-DREADD) was used as a control (Fig. 1a). Two weeks after injection, we detected Gi- and control-DREADD labelled with mCherry and EGFP, respectively, in gray matter at spinal level Th 8 (Fig. 1b). We revealed that DREADD expression was restricted to NeuN^+^ neurons, but not to GFAP^+^ astrocytes, in the dorsal column of the spinal cord (Fig. 1c).

To examine the effect of neuronal silencing with Gi-DREADD administered CNO, we generated a targeted EAE mouse model (Fig. 1d). We assessed EAE scores for 28 days after cytokine and pertussis toxin (PTX) injection. Neuronal silencing by Gi-DREADD attenuated motor deficits when compared with control-DREADD administered CNO (Fig. 1e). The maximum and accumulated EAE scores in Gi-DREADD mice were significantly lower than those in control-DREADD mice (Fig. 1f,g). These results demonstrate that neuronal silencing by Gi-DREADD alleviates motor symptoms in targeted EAE.

### Inhibitory DREADD suppresses pathological alterations and inflammatory response

**Figure 2:**
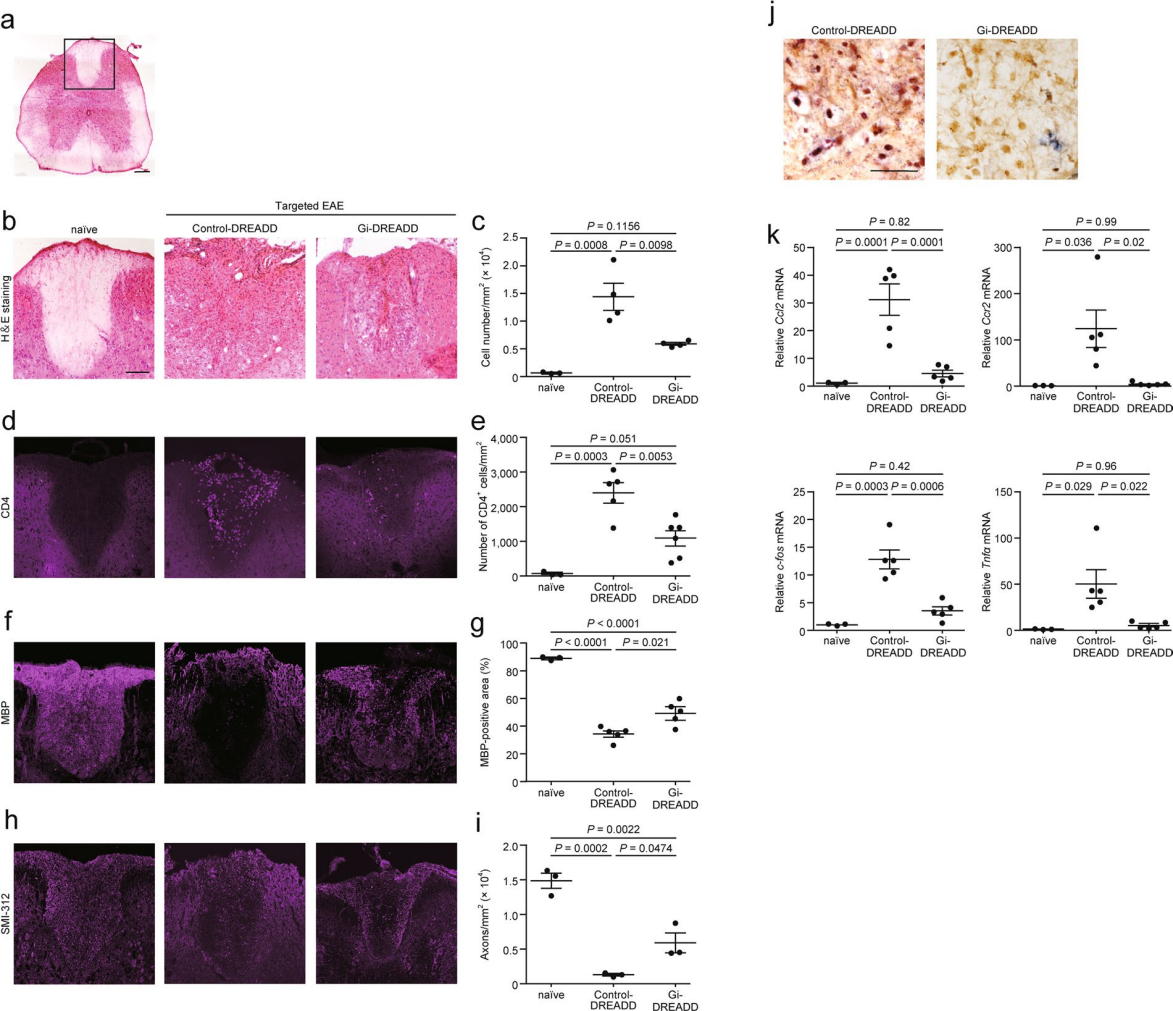
CNO-gated DREADD treatment suppressed infiltration of inflammatory cells, demyelination, and axonal degeneration in the dorsal column, and reduced the expression of chemokines and cytokines on day 17, at the time of peak EAE scores. (**a**) H&E staining of Th 8 level of spinal cord. A boxed region represents the dorsal column studied in (**b,d,f** and **h**). Scale bar: 200 μm. Representative images of (**b**) H&E staining, (**d**) CD4^+^ lymphocytes, (**f**) MBP, and (**h**) anti-pan-axonal neurofilament marker (clone: SMI-312) in the dorsal column of naïve and control- or Gi-DREADD-injected mice. Quantification of (**c**) cell number, (**e**) number of CD4^+^ lymphocytes, (**g**) MBP-positive area, (**i**) number of axons. n = 3–6. Scale bars: 100 μm. (**j**) Representative images of *in situ* hybridization of *Ccl2* (purple) and staining with NeuN (brown). (**k**) Expression of *Ccl2*, *Ccr2*, *c-fos*, and *Tnfα* mRNAs in the spinal cord of naïve and control- or Gi-DREADD-injected mice; n = 3, 5. Scale bars: 50 μm. Data are presented as mean ± sem. one-way ANOVA followed by Tukey comparison test.

We studied pathological alterations following Gi-DREADD administration to the dorsal column (Fig. 2a, boxed region) of targeted EAE mice on day 17 (peak of disease). Hematoxylin-eosin (H&E) staining showed that Gi-DREADD significantly reduced the number of inflammatory cells (Fig. 2b,c). When compared with control-DREADD, Gi-DREADD also significantly reduced the number of CD4^+^ lymphocytes (Fig. 2d,e), and accumulation of CD11b^+^ cells (Supplemental Fig. 1). Moreover, Gi-DREADD reduced the area of demyelination, as assessed with immunostaining for myelin basic protein (MBP), and axonal loss, as assessed by immunostaining for SMI-312, a neurofilament marker (Fig. 2f–i). *In situ* hybridization showed that *Ccl2* mRNA was detected in neurons stained with NeuN (Fig. 2j). Expression of *Ccl2* was reduced in spinal cord lesions from Gi-DREADD mice compared with control-DREADD mice (Fig. 2j). *Ccl2*, *Ccr2, c-fos*, and *Tnfα* mRNA levels in the lesions of control-DREADD mice were significantly upregulated compared with those of naïve mice, while Gi-DREADD mitigated this upregulation such that mRNA expression approximated to that of naïve mice (Fig. 2k).

### Neuronal silencing suppresses the migration of activated CD4+ lymphocytes

**Figure 3:**
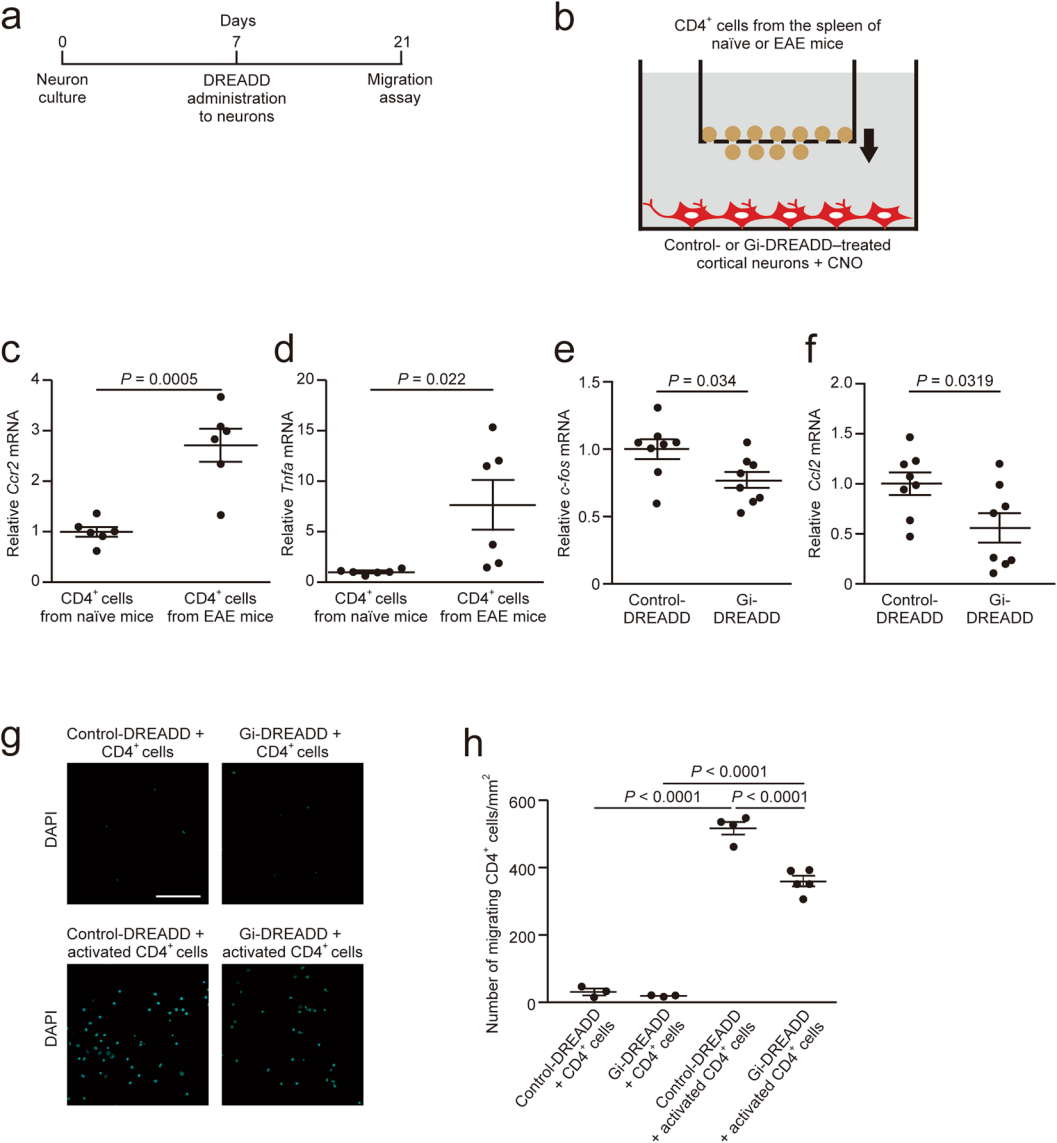
Inhibitory DREADD suppressed the migration of CD4^+^ lymphocytes towards neurons. (**a**) Experimental schema. Control- or Gi-DREADD-carrying-AAV9 was administered to embryonic cortical neurons placed in the lower chamber seven days after seeding of neurons. (**b**) Schematic illustration. Control- or Gi-DREADD-expressing cortical neurons and MACS-sorted splenic CD4^+^ lymphocytes from targeted EAE mice were incubated for 4 h at 37 °C. CNO (1 μM) was administered to the lower chamber 30 min prior to addition of CD4^+^ lymphocytes. mRNA expression of (**c**) *Ccr2* and (d) *Tnfα* in CD4^+^ lymphocytes (n = 6, Student’s *t-*test). mRNA expression of (**e**) *c-fos* and (**f**) *Ccl2* in cortical neurons (n = 8, Student’s *t-*test). (**g**) Representative images of DAPI-stained CD4^+^ lymphocytes on the lower filter surface in the migration assay. (**h**) Quantification of the number of migrating CD4^+^ lymphocytes (n = 3–5, one-way ANOVA followed by Tukey comparison test). Data are presented as mean ± sem. Scale bars: 25 μm.

To examine whether neuronal silencing suppresses the migration of CD4^+^ lymphocytes, we studied *in vitro* migratory activity of CD4^+^ lymphocytes isolated from the spleen of EAE mice (Fig. 3a). We counted CD4^+^ lymphocytes migrating towards embryonic cortical neurons infected with DREADD-carrying-AAV9 using the Transwell culture system (Fig. 3b). *Ccr2* and *Tnfα* expression in activated CD4^+^ lymphocytes from EAE mice was significantly upregulated compared with that in CD4^+^ lymphocytes from naïve mice (Fig. 3c,d). Gi-DREADD–treated neurons showed significant reductions in *c-fos* and *Ccl2* mRNAs when compared with control-DREADD–treated neurons (Fig. 3e,f). CCR2-expressing CD4^+^ lymphocytes isolated from EAE mice exhibited high migratory activity compared with CD4^+^ lymphocytes isolated from naïve mice (Fig. 3g,h). Gi-DREADD–treated neurons significantly reduced the migration potential of activated CD4^+^ lymphocytes from EAE mice (Fig. 3g,h). CCL2 binds sorely to CCR2^6^. Therefore, we hypothesize that neuronal CCL2 and lymphocytic CCR2 signaling potentiates CD4^+^ lymphocytes migration.

**Figure 4:**
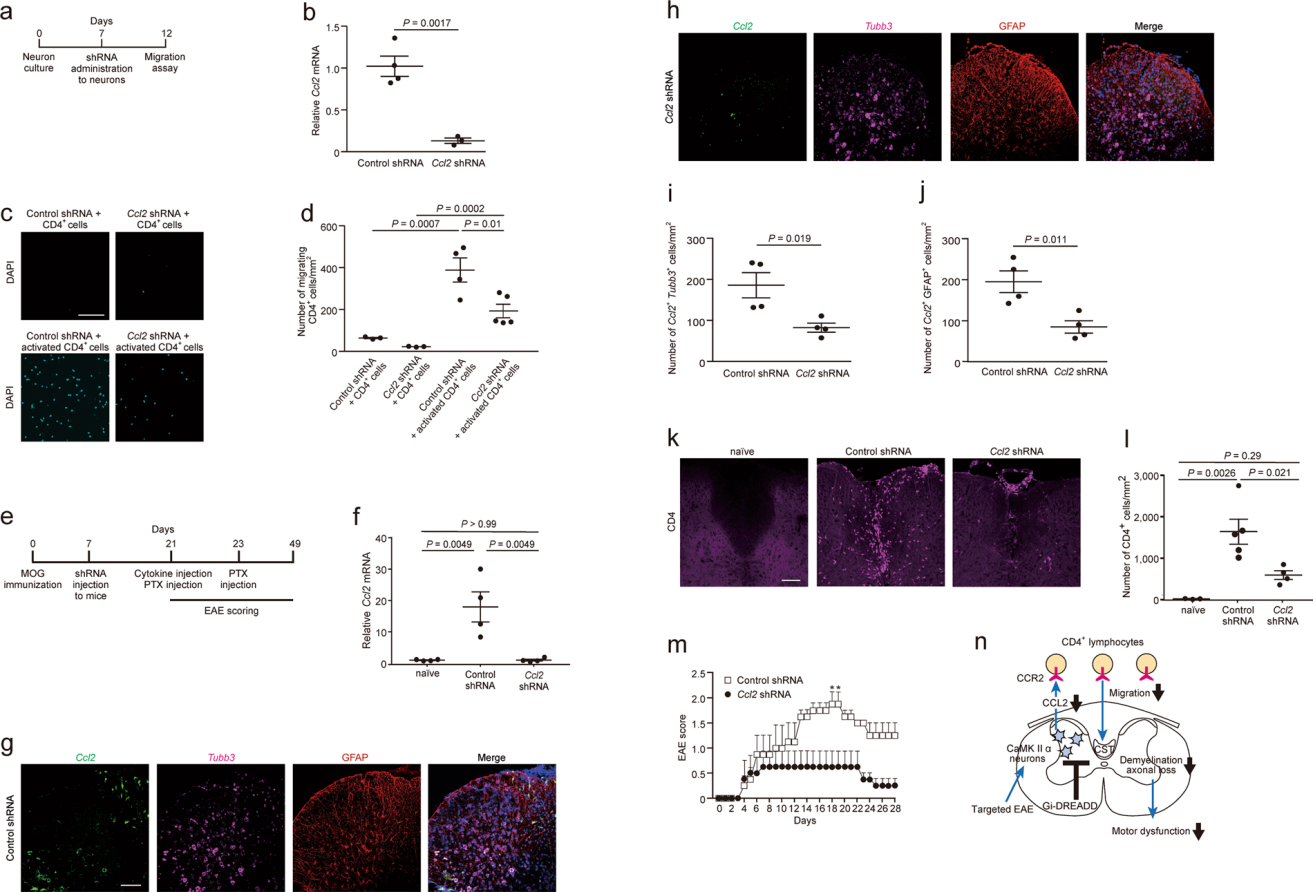
Suppression of *Ccl2* in targeted EAE neurons is a possible mechanism of mitigating EAE severity and reduction of CD4^+^ lymphocytes migration. (**a**) Schema of *Ccl2* shRNA experiment *in vitro*. *Ccl2* shRNA was administered to cultured cortical neurons five days before the migration assay. (**b**) *Ccl2* mRNA levels in cortical neurons (n = 3 and 4, Student’s *t-*test). (**c**) Representative images of DAPI-stained CD4^+^ lymphocytes on the lower filter surface in the migration assay and (**d**) quantification of CD4^+^ lymphocytes (n = 3–5, one-way ANOVA followed by Tukey comparison test). Scale bars: 25 μm. (**e**) Schema of *Ccl2* shRNA experiment *in vivo*. Seven days after MOG immunization, *Ccl2* shRNA-carrying-AAV9 was injected at spinal level Th 9. Cytokines were injected at the Th 8 spinal level 14 days after ShRNA administration. EAE scores were assessed from day 0 to 28 after cytokine injection. (**f**) *Ccl2* mRNA levels in the spinal cord of naïve and control- or *Ccl2* shRNA-injected mice; n = 3, 5. Representative images of fluorescent *in situ* hybridization of *Ccl2* (green) and *Tubb* (magenta), and staining with GFAP antibody (brown) and DAPI (blue) in the spinal cord of (**g**) control shRNA- or (**h**) *Ccl2* shRNA-injected mice. Scale bar; 100 μm. Quantification of (**i**) *Ccl2*-expressing neurons and (**j**) *Ccl2*-expressing astrocytes (n = 4, Student’s *t*-test). (**k**) Representative images of CD4^+^ lymphocytes in the dorsal column of naïve and control- or *Ccl2* shRNA-injected mice and (**l**) quantification of number of CD4^+^ lymphocytes (n = 3–5, one-way ANOVA followed by Tukey comparison test). Scale bar; 100 μm. (**m**) EAE scores (n = 4, two-way ANOVA followed by Sidak test). Data are presented as mean ± sem. (**n**) Hypothetical illustration. Excitation of CaMKIIα-expressing neurons of targeted EAE mice promotes CD4^+^ lymphocytes migration which herald demyelination, axonal loss, and consequently motor dysfunction (blue arrows). Neuronal CCL2 upregulated by neuronal excitation would be involved in lymphocytes migration. Neuronal silencing of CaMKIIα-expressing neurons suppresses CD4^+^ lymphocytes migration via reduction of neuronal CCL2 production and alleviates EAE pathology (black bar and arrows). CST, corticospinal tract.

Lastly, we assessed the possibility that neuronal CCL2 in targeted EAE is involved in the migration of CD4^+^ lymphocytes. We studied a migration assay with *Ccl2* knockdown *in vitro* by administration of *Ccl2* shRNA- or control shRNA-carrying-AAV9 (Fig. 4a). We found cortical neurons labelled with MAP2 occupied a major portion of *Ccl2* shRNA-expressing cells, and a small portion of *Ccl2* shRNA-expressing cells was astrocytes labelled with GFAP (Supplemental Fig. 2c,d,f). Similarly, the major portion of control shRNA was expressed in neurons (Supplemental Fig. 2a,b,e). *Ccl2* mRNA levels decreased to 12.8% by *Ccl2* shRNA treatment (Fig. 4b). *Ccl2* shRNA significantly reduced the migration of CD4^+^ lymphocytes towards embryonic cortical neurons (Fig. 4c,d). These results suggest that knockdown of *Ccl2* in neurons might reduce the migration of CD4^+^ lymphocytes *in vitro*. We next studied whether neuronal CCL2 promoted CD4^+^ lymphocytes migration *in vivo*. *Ccl2* knockdown was performed around EAE lesions by injection of *Ccl2* shRNA- or control shRNA-carrying-AAV9 into the spinal cord (Fig. 4e). *Ccl2* shRNA reduced the expression of *Ccl2* mRNA around the lesions (Fig. 4f). Fluorescent *in situ* hybridization of *Ccl2* with RNAscope probes showed that its expression levels in both neurons and astrocytes, labelled with *β-tubulin isotype III* (*Tubb3*) and GFAP, respectively, were downregulated by *Ccl2* shRNA treatment (Fig. 4g–j). *Ccl2* shRNA suppressed CD4^+^ lymphocytes accumulation *in situ* in the targeted EAE mice (Fig. 4k,l). Knockdown of *Ccl2* alleviated EAE severity compared with control shRNA (Fig. 4m). Taken together, neuronal CCL2 is thought to be involved in promoting CD4^+^ lymphocytes migration in targeted EAE mice.

## Discussion

Neuronal excitotoxity mediated by CaMKIIα causes neuronal damage in EAE^16^. In order to alleviate neuronal excitotoxity in the mouse model of MS, we applied Gi-DREADD combined with CNO administration to targeted EAE mice. We found that neuronal silencing in the targeted EAE mice improved EAE clinical scores by suppressing the migration of CD4^+^ lymphocytes and alleviated demyelination and axonal degeneration (Fig. 4n).

EAE is the most widely used animal model for MS; however, this model causes disseminated inflammatory lesions in the CNS^20^. This property makes it difficult to investigate the effect of regional intervention on inflammatory lesions of EAE. In the present study, we thus used targeted EAE induced by administration of myelin oligodendrocyte glycoprotein (MOG) and PTX and by stereotactic injection of cytokines to generate focal inflammatory lesions. We detected neuronal activation in the inflammatory lesions by *c-fos* expression. Activated T cells in the EAE inflammatory lesions stimulated extracellular glutamate release to enhance Ca^2+^ influx into neurons^14,21^. Ca^2+^ accumulation in neurons induced CaMKIIα activation, thereby promoting phosphorylation of the N-methyl-D-aspartate (NMDA) receptor and consequently causing neuronal excitotoxicity^22,23^. Inhibition of CaMKIIα activity in spinal cord neurons alleviates clinical symptoms in the EAE model^16^, suggesting a pathological role of excitatory neurons in EAE.

In the present study, neuronal silencing induced by inhibitory DREADD mitigated hind-limb paralysis, restored pathological alterations, and attenuated upregulation of *Ccl2*, *Ccr2, c-fos*, and *Tnfα* mRNAs in the spinal cord lesions of targeted EAE. The CCL2-CCR2 pair is critical for expression of EAE^3,5,6^. CCL2 is also produced in embryonic cortical neurons of mice and functions in neuron signaling and development^24^. CCL2 has been shown to attract CCR2-expressing encephalitogenic T cells in migration assays^6,25^. Therefore, we used embryonic cortical neurons to study the activity of neuron-derived CCL2 in a migration assay. During *in vitro* experiments, embryonic cortical neurons attracted CCR2-expressing CD4^+^ lymphocytes from EAE mice. Both administration of Gi-DREADD–carrying-AAV9 and *Ccl2* shRNA treatment to embryonic cortical neurons suppressed CD4^+^ lymphocytes migration. Downregulation of neuronal CCL2 would suppress CD4^+^ lymphocytes migration. Several studies revealed that CCL2 attracted CCR2-expressing encephalitogenic T cells in migration assays^6,25^, and neuronal CCL2 promoted migration of immune cells^7,8^. Activated neurons would attract inflammatory lymphocytes via CCL2 upregulation, and contribute to EAE pathogenesis. In the present fluorescent *in situ* hybridization study, *Ccl2* shRNA treatment reduced the expression of *Ccl2* in both neurons and astrocytes in the spinal cord of targeted EAE mice. A previous study clarified the involvement of astrocytic CCL2 to pathophysiology of EAE^13^. We thus assume that astrocytic CCL2 also potently promotes CD4^+^ lymphocytes migration in target EAE mice.

Previous studies demonstrated that systemic administration of TNFα and local delivery of TNFα by retrovirus-transduced T lymphocytes exacerbated EAE^26,27^. By contrast, anti-TNFα antibody reduced the severity of EAE^28^. TNFα administration upregulated CCL2 expression in cortical neurons^29^. In the current study, activated CD4^+^ lymphocytes exhibiting high migration activity enhanced TNFα expression. TNFα is also thought to be involved in disease process on EAE.

According to the results of H&E staining and CD4 immunostaining in the current study, CD4^+^ lymphocytes were a part of cells accumulated in targeted EAE lesion. Targeted EAE lesions show accumulation of T cells, macrophages, microglia, and astrocytes^20^. We found that accumulation of CD11b^+^ cells was reduced after Gi-DREADD in the spinal cord of targeted EAE mice. We assume that neuronal silencing could also reduce accumulation of these immune cells. EAE is initiated when activated autoreactive T cells cross the blood-brain barrier to reach the CNS^6,9^. Deletion of CD4^+^ lymphocytes by anti-CD4 antibody administration completely protected EAE mice from development of the disease^30^^.^ We recognize that neuronal silencing by Gi-DREADD targets on attenuation of lymphocytes infiltration into the CNS at the early stage of the disease.

Neuronal silencing by inhibitory DREADD significantly, but incompletely, mitigated the EAE disease process. Astrocytes and microglia, as well as neurons, are involved in the pathogenesis of EAE. Astrocytes promote immune cell trafficking, leading to release of pro-inflammatory chemokines (CCL2, CCL4, CCL5, CCL20, CXCL9, and CXCL10), cytokines (TNFα, IFNγ, IL-6, IL-12, and IL-23), and glutamate^31,32^. Microglia also contribute to EAE by releasing chemokines (CCL2, CCL3, CCL4, CCL5, CCL12, and CCL22) and cytokines (TNFα, IL-1, IL-6, IL-12, and IL-23)^33^. Inhibitory DREADD mitigated upregulation of *Ccl2* and *Ccr2* mRNAs in EAE lesions to levels equivalent to naïve mice; however, CD4^+^ lymphocyte infiltration and EAE symptoms were only partially improved. In addition to CCL2, chemokines CCL7 and CCL8, which activate CCR2-expressing CD4^+^ lymphocytes^3,34^, are upregulated in the CNS of EAE mice. The migration of CD4^+^ lymphocytes is also induced by other chemokine receptor-ligand interactions, such as CCR6-CCL20^35^, CXCR3-CXCL9, 10, and 11; CXCR4-CXCL12; and CCR7-CCL19 and 21, with all these chemokine receptors and chemokines being upregulated in EAE^36–39^. These redundant, complex chemokine systems could explain the significant but incomplete improvements in CD4^+^ lymphocyte migratory activity and clinical defects observed in this study with CCL2 deletion.

To our knowledge, we for the first time adopted inhibitory DREADD and a migration assay using embryonic cerebral cortex neurons with CD4^+^ T cells. This methodology will pave a new way to investigate the pathophysiological role of excitatory neurons in EAE.

## Materials and methods

### Animals

Eight-week-old male C57BL/6 J mice (Japan SLC) were used in this study. All experimental procedures were approved by the Institutional Animal Care Committee of Osaka University and complied with the guidelines for the care and use of laboratory animals at Osaka University.

### Recombinant AAV9 vector production

To produce the vector for DREADD, we used pAAV-CaMKIIα-hM4D (Gi)-mCherry. pAAV-CaMKII-EGFP was used as a control construct. pAAV-CaMKIIα-hM4D (Gi)-mCherry was a gift from Bryan Roth (Addgene plasmid # 50477; http://n2t.net/addgene:50477; RRID: Addgene_50477), and pAAV-CaMKII-GFP was a gift from Edward Boyden (Addgene plasmid # 64545; http://n2t.net/addgene:64545; RRID: Addgene_64545). Procedures were performed according to a previous study^40^. Briefly, 300 µg pAAV-CaMKIIα-hM4D (Gi)-mCherry or 300 µg pAAV-CaMKIIα-EGFP, 300 µg Rep/Cap plasmid, or 600 µg adenovirus-helper plasmid was transfected into cultured 293 AAV cells. Five days after transfection, we collected cells and isolated rAAV. The genomic titers were measured using SYBR Green Master Mix (Thermo Fisher Scientific). ITR primers were obtained from AAV^®^pro titration kit (TaKaRa). Genomic titers were adjusted to 1 × 10^11^ gc/µl.

### Induction of targeted EAE with DREADD

Targeted EAE was generated according to a previous study^1^. Seven days after MOG_35–55_ immunization, we performed dorsal laminectomy under anesthesia and injected Gi- or control-DREADD-carrying rAAV9 (1.5 μl, 1.5 × 10^11^ gc) at spinal level Th 9. Twenty-one days after injection, we administered a cytokine mixture containing TNFα (750 ng, R&D Systems) and IFNγ (2,500 U, Peprotech) at spinal level Th 8. On the same day, we implanted an osmotic pump containing clozapine N-oxide (CNO, 4 mg/ml in PBS, Enzo Life Sciences). CNO was infused for 14 days at a flow rate of 2 μg/h. We intravenously administered pertussis toxin (200 ng, List Biological Laboratories) immediately prior to and 48 h after cytokine injection. We assessed daily EAE clinical scores according to the following criteria^1^: 0, no clinical disease; 0.5, partial tail weakness or slight loss of muscle tone; 1, tail weakness; 1.5, loss of tail reflex with hind leg inhibition without gait abnormality; 2, partial hind limb paralysis with gait abnormality; 3, complete hind limb paralysis; 4, front and hind limb paralysis; and 5, moribund state.

### Histological analysis

Procedures were performed according to previous studies^1^. Tissues were cut into 25-μm-thick sections using a cryostat. Hematoxylin-eosin (H&E) staining was performed as discussed elsewhere^1^. For immunohistochemistry, sections were incubated in 5% goat serum in PBS with 0.1–0.3% Triton-X for 1 h at room temperature (RT), and then incubated with primary antibody in 5% goat serum overnight at 4 °C. Anti-NeuN, anti-GFAP, anti-CD4, anti-CD11b, anti-MBP, anti-SMI-312, and anti-MAP2 as in Table S1 were used. The sections were incubated with the following secondary antibodies for 1 h at RT in the dark: Alexa-Fluor 488-, 568-, or 647-conjugated secondary antibodies (Table S1). The nuclei were stained with 4’, 6-diamidino-2-phenylindole (DAPI, 1:1000, Dojindo Laboratories) for 10 min at RT.

The images of H&E-stained sections were acquired with bright field microscopy (Olympus, IX83), while those of immunohistochemical studies were acquired with confocal microscopy (Olympus, FV3000). All images were analyzed using ImageJ software (National Institutes of Health).

### *In situ* hybridization

The animals were perfused with diethylpyrocarbonate (DEPC)-treated ice-cold PBS, followed by DEPC-treated 4% PFA under anesthesia. The tissues were fixed with 4% PFA for 3 days at 4 °C and subsequently transferred to DEPC-treated 30% sucrose in DEPC-treated PBS overnight at 4 °C. Tissues were cut into 25-μm-thick sections using a cryostat. The primer sequence for the *Ccl2* RNA probe is shown in Table S2. We integrated PCR product into pCR^TM^-Blunt II-TOPO^®^ (Invitrogen). DNA templates were digested with BamH1 or Xho1 for 3 h at 37 °C. Linearized DNA was labelled with DIG RNA labelling mix (Roche) and transcribed by T7 or SP6 RNA polymerase (Roche) for 2 h at 37 °C, followed by lithium chloride precipitation.

Sections were treated with proteinase K for 10 min at RT and acetylated with 0.1 M triethanolamine and 0.25% acetic anhydride for 10 min at RT. After immersion with hybridization buffer for 1 h at RT, the sections were hybridized with 500 ng *Ccl2* RNA probe in hybridization buffer for 16 h at 63.5 °C. After washing these sections, they were placed in 20 mM iodoacetamide for 20 min at RT. The sections were immersed with blocking solution (10% blocking reagent [Roche], 0.1 M Tris-HCl [pH 7.6], 0.15 M NaCl, 0.00075% tween, 20, 4% normal lamb serum) for 30 min at RT. Next, the sections were reacted with alkaline phosphatase-conjugated anti-DIG antibody in blocking solution for 90 min at RT and then incubated with 4-nitro blue tetrazolium chloride (NBT, 5 μl/ml, Roche) and 5-bromo-4-chloro-3-indolyl-phosphate (BCIP, 3.75 μl/ml, Roche) for 1–3 days at 4 °C. Sections were subsequently incubated with mouse anti-NeuN (Table S1) in PBS overnight at 4 °C, and then biotinylated goat anti-mouse IgG antibody for 30 min at RT. Finally, sections were incubated with Elite^®^ ABC Reagent (Vector laboratories) for 1 h at RT for biotin-avidin reaction. Peroxidase detection was performed with 0.2 mg/ml 3, 3’-diaminobenzidine (DAB, FUJIFILM) in TBS buffer (50 mM Tris-HCl with 0.85% NaCl) containing 0.01% H_2_O_2_ for 3 min at RT. The sections were fixed with fixation solution (10% formaldehyde, 0.1% glutaraldehyde, 1 × PBS) for 15 min at RT. After dehydration, the sections were observed under bright field microscopy (Olympus, IX83).

### Cortical neuron culture

Cortical neurons were isolated from the cerebral cortex of E18 C57 BL/6 J mice and cultured following the method described in our previous study^40^ with minor modifications. First, a 24-well plate was precoated with 10 mg/ml poly-L-lysine (PLL, Sigma) overnight at 37 °C. After cortical neurons were dissociated to single cells, 5 × 10^5^/ml neurons were suspended in Neurobasal^®^ medium (Gibco) containing 2% B27 supplement (Gibco) and 1% penicillin-streptomycin (Gibco) and cultured in a PLL-coated 24-well plate. Purity of the neurons (90.0%) was determined by MAP2 staining. After 7 days, cultured neurons were infected with rAAV9-carrying-Gi- or -control-DREADD.

### Isolation of CD4^+^ lymphocytes from the spleen

Targeted EAE mice were anesthetized and perfused with ice-cold PBS. The spleen was dissected and underwent hemolysis. CD4^+^ lymphocytes were collected using CD4^+^ T cell isolation kit (Myltenyi) according to the manufacturer’s protocol. Subsequently, 5 × 10^6^/ml cells were suspended in RPMI-1640 (Gibco) containing 2% FBS and 1% penicillin-streptomycin.

### Migration assay and cytological analysis

An illustration of the experiment is shown in Fig. 3b. To silence neuronal activity, CNO (1 μM in medium) was added to cultured neurons for 30 min at 37 °C. Then, 5 × 10^5^ CD4^+^ lymphocytes isolated from the spleen in 100 μl of medium were transferred to a 5 μm pore size cultured insert (Corning) precoated with 50 μg/ml of fibronectin. Cortical neurons and CD4^+^ lymphocytes were incubated for 4 h at 37 °C, and then CD4^+^ lymphocytes on the lower filter surface were fixed with 4% PFA for 30 min at RT. Fixed cells were treated with 0.1% Triton-X and stained with DAPI. Four independent fields were observed with fluorescence microscopy (Olympus, BX5).

### Quantitative polymerase chain reaction (qPCR)

We analyzed gene expression of spinal cord tissues in targeted EAE mice and in cultured cortical neurons collected after the migration assay. The procedures were performed as described in a previous study^40^. The primer sequences are described in Table S2^41–44^. The relative expression was normalized to 18S rRNA.

### shRNA experiment

A pair of 60-nucleotide oligonucleotides encoding the 19-nucleotide *Ccl2* shRNA (target sequence: 5′-GAAGTTGACCCGTAAATCT-3′)^8^ and turbo red fluorescent protein were designed and cloned into BamH1 and EcoR1 sites of the pAAV-MCS plasmid (Stratagene). *Ccl2* shRNA-expressing AAV9 was generated according to the method prepared for DREADD. Throughout the *in vivo* experiment, the methods for generating targeted EAE and measurement of clinical scores were the same as those used in the DREADD experiment. *Ccl2* or control shRNA-carrying-AAV9 was injected at the Th 9 spinal level 14 days after induction with MOG_35–55_. For the *in vitro* experiment, *Ccl2* shRNA was administered to cortical neurons isolated from E18 mice 5 days before performing the migration assay. The number of migrating CD4^+^ lymphocytes was assessed with confocal microscopy (Olympus, FV3000). Successful downregulation of *Ccl2* expression was examined by RT-PCR in shRNA-treated neurons.

### Fluorescent *in situ* hybridization

We used RNAscope probes (*Ccl2* 311791; *Tubb3* 423391-C4) designed by Advanced Cell Diagnostics (ACD), and stained four 16-μm cryosections from the spinal cord of mice with ACD RNAscope kits according to manufacturer’s instructions.

### Statistics

Results are expressed as mean ± sem. Significance was measured with unpaired *t* tests, one-way ANOVA followed by the Tukey test, or two-way ANOVA followed by the Sidak or Bonferroni tests. A *P* value of <0.05 was considered statistically significant. Statistical analyses were conducted using GraphPad Prism 7 (GraphPad software).

## Supplementary information

Supplementary figures and tables.
