# Supplementary material for: Neurons promote encephalitogenic CD4+ lymphocyte infiltration in experimental autoimmune encephalomyelitis
Source: Sci Rep. 2020 Apr 30;10:7354. doi: 10.1038/s41598-020-64363-z (PMC7192891; doi:10.1038/s41598-020-64363-z)
Supplement: Supplementary file 1 — Supplementary figures and tables. [file 41598_2020_64363_MOESM1_ESM.pdf]

Supplementary Information

Neurons promote encephalitogenic $CD4^+$ lymphocyte infiltration in experimental autoimmune encephalomyelitis

Author names and affiliations

Yuki Nakazato[1, 3], Yuki Fujita[1, 2], Masamitsu Nakazato[3], and Toshihide Yamashita[1, 2, 4, 5]

1. Department of Molecular Neuroscience, Graduate School of Medicine, Osaka University, Osaka, Japan.
2. WPI Immunology Frontier Research Center, Osaka University, Osaka, Japan.
3. Department of Internal Medicine, Division of Neurology, Respirology, Endocrinology, and Metabolism, Department of Internal Medicine, Faculty of Medicine, University of Miyazaki, Miyazaki, Japan. [4]
4. Graduate School of Frontier Bioscience, Osaka University, Osaka, Japan.
5. Department of Neuro-Medical Science, Graduate School of Medicine, Osaka University, Osaka, Japan

Corresponding author

Toshihide Yamashita

Tel.: 81-6-68793661; Fax: 81-6-68793669; E-mail: yamashita@molneu.med.osaka-u.ac.jp

Supplementary Tables

Table S1. Antibodies for immunohistochemistry (IHC)

Antibodies	Origin (Cat#)	Dilution	Incubation period
Mouse anti-NeuN	Millipore (MAB377)	1:500 (IHC)	Overnight
		1:100 (ISH)	Overnight
Mouse anti-GFAP	Sigma-Aldrich (G3893-. 2ML)	1:500	Overnight
Rat anti-CD4	BD Pharmingen (550278)	1:200	Overnight
Rat anti-CD11b	BD Pharmingen (550282)	1:500	Overnight
Rat anti-MBP	Abcam (ab7349)	1:500	Overnight
Mouse SMI-312	BioLegend (837904)	1:500	Overnight
Mouse anti-MAP2	Sigma-Aldrich (M4403-. 2ML)	1:500	Overnight
Goat anti-Rat AlexaFluor 488 secondary	Invitrogen (A11006)	1:1,000	1 h
Goat anti-Rat AlexaFluor 568 secondary	Invitrogen (A11077)	1:1,000	1 h
Goat anti-Mouse AlexaFluor 647 secondary	Invitrogen (A21236)	1:1,000	1 h
Sheep anti-Digoxigenin-AP	Roche (11093274910)	1:500	90 min
Biotinylated goat anti-mouse IgG antibody	Vector laboratories (BA-9200)	1:200	30 min

Table S2. The sequences of the oligonucleotides for qPCR, *in situ* hybridization (ISH) and shRNA experiment

Gene	Experiment		Sequence	Reference
Ccl2	qPCR	Forward	5′-TGGCTCAGCCAGATGCAGT-3′	41
		Reverse	5′-TTGGGATCATCTTGCTGGTG-3′	41
Ccr2	qPCR	Forward	5′-GTTACCTCAGTTCATCCA-3′	41
		Reverse	5′-CAAGGCTCACCATCATCGTAGTC-3′	41
Tnfα	qPCR	Forward	5′-GCACAGAAAGCATGATCCG-3′	41
		Reverse	5′-GCCCCCCATCTTTTGGG-3′	41
c-fos	qPCR	Forward	5′-CCGAATTCTTCCCCAAACTTCGACC-3′	42
		Reverse	5′-TAGAATTCGGCTGCCTTGCCTTCTC-3′	42
18S rRNA	qPCR	Forward	5′-GCAATTATTCCCCATGAACG-3′	43
		Reverse	5′-GGCCTCACTAAACCATCCAA-3′	43
Ccl2	ISH	Forward	5′-CCGTAATACGACTCACTATAGGGCAGGTGTCCCAAAGAAGCTG-3′	8
		Reverse	5′-CCGATTTAGGTGACACTATAGAATGGATTCACAGAGAGGGAAAA-3′	8
Ccl2	shRNA	Sense	5′-GATCTCCGAAGTTGACCCGTAAATCTTTCAAGAGAAGATTTACGGGTCAACTTCTTTTT-3′	44
		Antisense	5′-GATCTCCTGAGTAGGCTGGAGAGCTATTCAAGAGATAGCTCTCCAGCCTACTCATTTTTG-3′	44

Supplemental Fig. 1

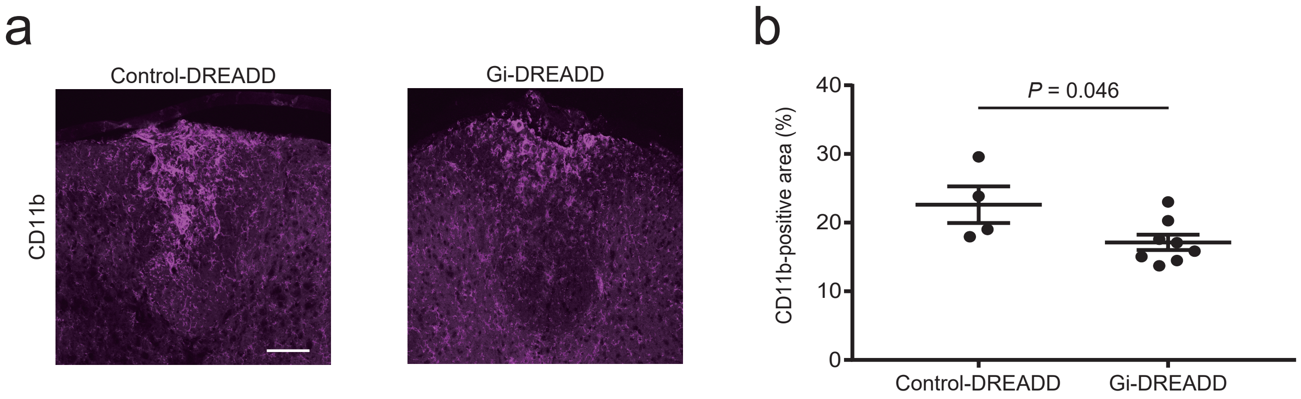

Supplemental Figure 1. CNO-gated DREADD treatment suppressed accumulation of CD11b-positive cells in the dorsal column.

(a) Representative images of CD11b-positive cells in the dorsal column of control- or Gi-DREADD–injected mice.

(b) Quantification of CD11b-positive area (n = 4–8, Student's *t*-test).

Data are presented as mean ± sem. Scale bars: 100 μm.

Supplemental Fig. 2

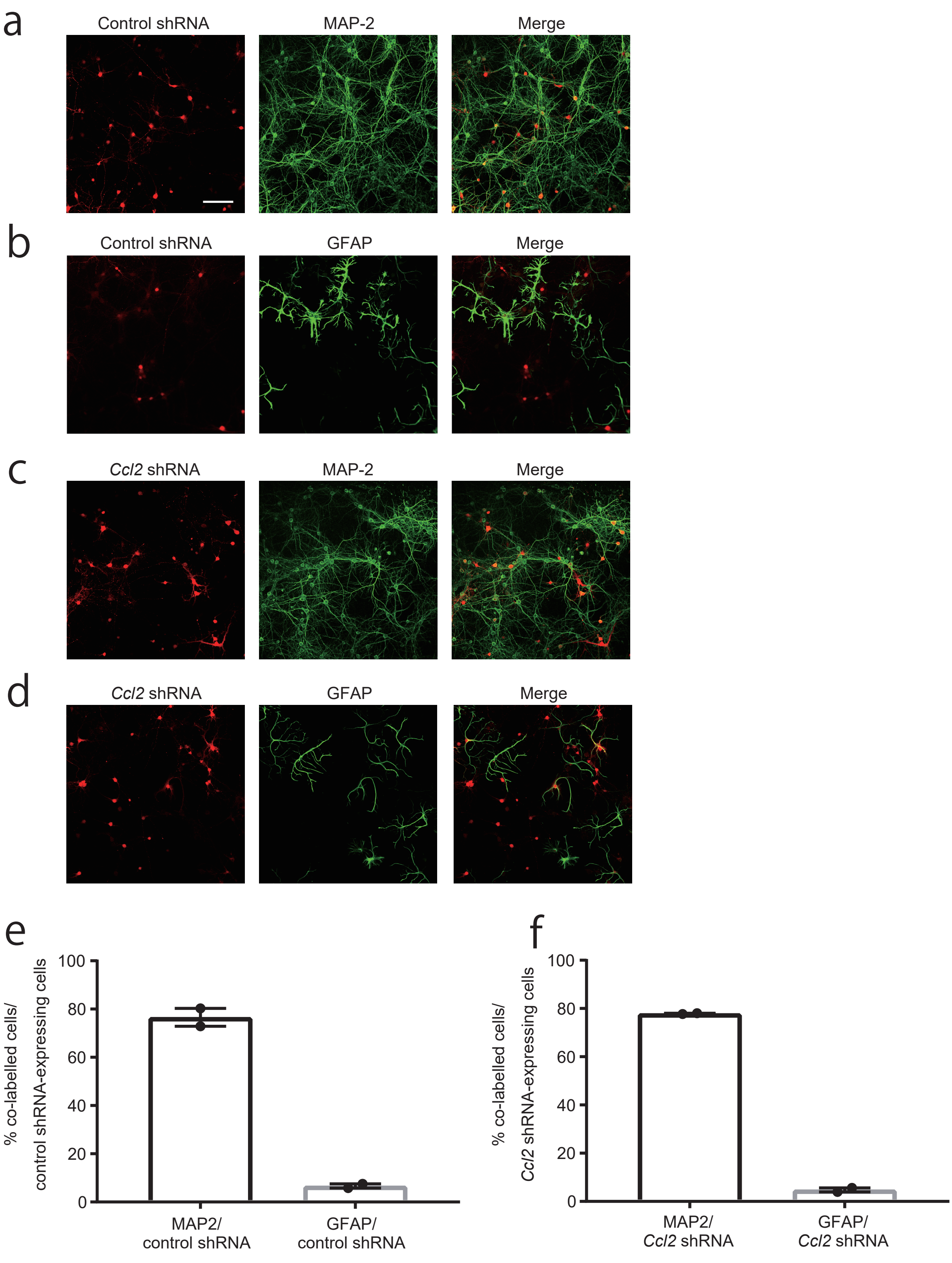

Supplemental Figure 2. *Ccl2* and control shRNA colocalization with MAP2 or GFAP *in vitro*. Control shRNA (red) colocalization with (a) MAP2 (green), and (b) GFAP (green) in the neuronal cultures. *Ccl2* shRNA (red) colocalization with (c) MAP2 (green), and (d) GFAP (green). Percentage of (e) control shRNA-expressing cells co-labelled with MAP2 or GFAP and (f) *Ccl2* shRNA expressing cells co-labelled with MAP2 or GFAP. n = 2. Data are presented as mean ± sem. Scale bar: 100 μm.
